# Radiographic Rate and Clinical Impact of Pseudarthrosis in Spine Radiosurgery for Metastatic Spinal Disease

**DOI:** 10.7759/cureus.3631

**Published:** 2018-11-25

**Authors:** Michael Zhang, Geoff Appelboom, John K Ratliff, Scott G Soltys, John R. Adler, Jon Park, Steven D. Chang

**Affiliations:** 1 Department of Neurosurgery, Stanford University School of Medicine, Stanford, USA; 2 Department of Radiation Oncology, Stanford University School of Medicine, Stanford, USA; 3 Department of Radiation Oncology, Stanford University Medical Center, Stanford, CA, USA; 4 Department of Neurosurgery, Stanford University Medical Center, Stanford, USA

**Keywords:** pseudarthrosis, hardware failure, stereotactic radiosurgery, spinal column metastases

## Abstract

Purpose

Pseudarthrosis within the spine tumor population is increased from perioperative radiation and complex stabilization for invasive and recurrent pathology. We report the radiographic and clinical rates of pseudarthrosis following multiple courses of instrumented fusion and perioperative stereotactic radiosurgery (SRS).

Methods

We performed a single institution review of 418 patients treated with non-isocentric SRS for spine between October 2002 and January 2013, identifying those with spinal instrumentation and greater than six months of follow-up. Surgical history, radiation planning, and radiographic outcomes were documented.

Results

Eleven patients who met criteria for inclusion underwent 21 sessions of spinal SRS and 16 instrumented operations. Radiographic follow-up was 48.9 months; 3/11 (27%) were with radiographic hardware failure, and one (9%) separate case ultimately warranted externalization due to tumor recurrence. SRS was administered to treat progression of disease in 12/21 (57%) procedures, and residual lesions in 7/11 (64%) procedures. Following first and second SRS, 8/11 (73%) and 2/7 (29%) patients were with symptomatic improvement, respectively.

Conclusion

Risk of pseudarthrosis following SRS for patients with oncologic spinal lesions will become increasingly apparent with the optimized management of and survival from spinal pathologies. We highlight how the need for local control outpaces the risk of instrumentation failure.

## Introduction

The management of spinal column metastasis has increasingly moved towards less invasive separation surgery followed by adjuvant stereotactic radiosurgery (SRS). This decompressive approach reduces the post-operative recovery burden while preserving high levels of functional independence [1, 2]. However, aggressive surgical stabilization and construct extension is still warranted among destabilizing and multiply-recurrent pathologies for preservation of function. The advantages and disadvantages of these large spinal constructs in already poor quality bone subject to further irradiation, become important in the pre-operative discussion.

Despite its radiobiological stressors, SRS is with known benefits toward pain and disease control [3-5]. The movement from adjuvant external radiation therapy (XRT) to SRS for spinal column metastasis provides higher doses with steeper drop-offs limiting direct radiation to the bony matrix and spinal cord [1, 6, 7]. Nevertheless, the long oncologic course for these patients exposes them to multiple treatment sessions and multiple modalities of radiation that additively undermine desirable fusion. Concerns for decreased periosteal osteoblastic proliferation, decreased vascularity, and increased bony pliancy subject patients to the risks of hardware failure, including implant migration, fusion failure, and biomechanical destabilization-associated pain that can warrant surgical revision [8, 9].

Unfortunately, the reduced life expectancies and post-operative tolerances of the overall spinal tumor population has made an assessment of post-SRS pseudarthrosis difficult. Furthermore, published series are limited by their partial reporting of radiation treatment history or follow-up, leading to a wide range of pseudarthrosis rates that are difficult to compare. Thus, the collective impact of serial, surgical and adjuvant radiation treatments on surgical decision-making is absent. Therefore, we assessed patients with perioperative SRS for recurrent spinal column metastasis necessitating instrumentation to qualify how the rates of radiographic and clinical pseudarthrosis guide their oncologic management.

## Materials and methods

Patient population

A retrospective review of a prospectively collected database of patients treated with non-isocentric SRS between October 2002 and May 2013 was performed. We limited assessment to patients with instrumented fusion with post-surgical SRS radiation, and at least six months of radiographic follow-up. From a total of 418 patients who were treated with SRS, 42 (10%) were with prior surgery and instrumentation, 35 (8.4%) with Karnofsky performance score (KPS) > 70, and 11 (2.6%) patients who met criteria for inclusion.

Primary outcome measured for the rate hardware failure, including radiographic and clinical pseudarthrosis, as well as the rate of operative externalization. Demographic and risk factors including sex, age at treatment, weight, smoking history, osteoporosis, primary disease histology, and KPS at time of index SRS treatment were recorded. All aspects of the study were performed in accordance with relevant guidelines and regulations as were approved by the Stanford Institute Review Board. Written informed consent was not required given the retrospective study format.

Treatment

Surgical and SRS treatment history were characterized for analysis. All instrumented and non-instrumented fusions to the spine were documented. Individual surgical interventions were further assessed for indication, need for instrumentation, time from any preceding SRS or instrumented surgery, location relative to prior surgery, symptoms prior to treatment, response to treatment, surgical approach as well as incorporation of corpectomy, junctional levels, and graft.

Regarding radiation treatment, all sessions with exposure to the spine by either conventional radiation therapy or SRS were documented. Individual SRS interventions, which were all performed at the authors’ institution, were further assessed for indication, levels treated, time from index instrumented-surgery, time from prior SRS, location relative to prior SRS, symptoms prior to SRS, and response to treatment. Biological equivalent doses were estimated with an alpha/beta of 10.

Statistical analyses

Differences in means and proportions were determined by Mann-Whitney/un-paired t-test. Probabilistic univariate and multivariate analyses were performed by logistic regressions. Statistical analyses were performed using Stata/SE 13.1 Software (StataCorp, College Station, TX, USA). Statistical significance was targeted for p < 0.05.

## Results

For the 11 patients who met inclusion criteria, mean radiographic follow-up was 48.9 (6-121) months. Assessment of primary outcomes identified four (36%) patients with hardware failure, within which 3/11 (27%) were with radiographically confirmed pseudarthrosis (Table 1). Among these patients, externalization was performed in one (9%) patient for screw loosening, which ultimately was attributed to tumor involvement. Clinically attributed neck pain was reported in 1/11 (9%), but no pseudarthrosis was detected and no revision was performed. Individual risk factors included 1/11 (9%) patients with osteoporosis, 4/11 (36%) with former smokers (none current), and 7/11 (64%) with elevated body mass index (BMI). All patients underwent chemotherapy after SRS, but 5/11 (45%) had not initiated a regimen beforehand. Two (18%) patients were with protracted steroid treatment. Three (27%) patients had unfavorable histologies, including one with lung and two with prostate primaries. The remaining patients were treated for breast, melanoma, thyroid, thymoma, and multiple myeloma [7].

**Table 1:** Surgical instrumentation, peri-operative timeline, and fusion features. CK: CyberKnife; F: Female; Gy: Gray; M: Male; NA: Not applicable; NSCLC: Non-small cell lung cancer; mo: month; RCC: Renal cell carcinoma; yr: year.

Case	Sex	Histology	Instrument Levels	Procedure	Indication	Disposition	Age at 1^st^ CK (yr)	Time to CK from 1^st^ Instrument (mo)	Time to 2^nd^ CK from 1^st^ CK (mo)	Time to 2^nd^ Surgery from 1^st^ Instrument (mo)	Graft	Hardware Failure	Radiographic Follow-up (mo)
1	F	NSCLC	C5-7	Corpectomy, ACDF	Unstable	NA	57	21	13	34	Allograft	Pseudarthrosis: After all procedures	34
			C5-6	Corpectomy Foraminotomy	Compression	Home					Unreported		
2	F	Breast	O-C4	Lateral mass osteotomy, Posterior fusion	Unstable	Home	68	0	61		Both	Pseudarthrosis: After index instrumentation and adjuvant SRS	56
3	F	Breast	C1-4	Posterior fusion	Unstable	Home	58	2			Allograft	N	56
4	M	RCC	L2-4	Corpectomy, Posterior fusion	Unstable	Home	59	6	5	18	Both	Externalized: At second instrumentation	30
			T10-S1	Posterior fusion, Removal of prior instrumentation	Compression	Home					Autograft		
5	M	RCC	T10-12 T8-L2	Corpectomy, Anterior and Poster Fusion,	Unstable	Rehab.	66	0	10		Allograft	N	34
6	M	Melanoma	NA	[Laminectomy]	NA	NA	56	3	7	11	NA	N	19
			C7-T9	Posterior fusion	Compression	Home					Unreported		
			C5-T8	Posterior fusion	Compression	Home					Allograft		
			T3-T5	Corpectomy, Posterior fusion	Compression	Home					Allograft		
7	M	Melanoma	NA	[Aborted resection]	NA	NA	49	1	14	9	NA	N	21
			T2-T7	Posterior fusion	Compression	Home					Both		
			T3-T7	Corpectomy, Anterior and Posterior fusion	Compression	Home					Both		
			NA	[Laminectomy]	NA	Rehab.							
8	M	Melanoma	NA	[Uninstrumented resection]	NA	NA	74	6	4		NA	N	62
			NA	[Uninstrumented resection]	NA	NA					NA		
			C7-T2	Posterior fusion	Compression	Home					Both		
9	F	Thyroid	T4-T8	Corpectomy, Posterior fusion	Unstable	Home	64	16	44		Allograft	Pseudarthrosis: After all procedures	121
10	M	Thymoma	T6-T10	Corpectomy, Posterior fusion	Compression	Home	41	1			Allograft	N	6
			NA	[Laminectomy]	NA	NA					NA		
11	M	Multiple Myeloma	T4-T8	Posterior fusion	Compression	Home	34	38	61		Allograft	N	99

There were a total of 21 sessions of non-isocentric hypofractionated treatment of spinal SRS, all treated by CyberKnife at the authors’ institution, and 12 sessions of spinal RT (Table 2). After index SRS, nine (82%) patients underwent repeat SRS and one (9%) patient required a third session. Time to SRS after first instrumented surgery was median three (0-38) months. A mean 1.0 (1-3) and 2.5 (1-3) levels were treated at first and second SRS, respectively. Median time to second SRS was 13.0 months (4-61). Among the eight repeat treatment plans, one took place at a distinctly separate spinal distribution. SRS treatment plans were with median 24 Gy (16-35) prescription dose, in median three fractions (1-5) to the 80% (70-84) isodose line with a median maximum dose of 29.3 Gy (20.5-43.8). A median 23.5 cc (0.77-247) was treated with a median conformity index of 1.35 (1.1-2.04). Notably, the biological equivalent dose (BED) for those without instrumentation failure was higher, with median BED for those with and without failure at 51.3 (40-70.4) Gy vs 42.4 (35.7-50.4) Gy (p = 0.0051), respectively.

**Table 2:** Radiation planning and radiation history. cc: Cubic centimeter; CI: Conformity index; CK: CyberKnife; Gy: Gray; IMRT: Intensity-modulated radiation therapy; NA: Not applicable; mo: month; yr: year.

Case	CK Session	Indication	SRS Target	Prescription Dose (Gy)	Fractions	Max Dose (Gy)	Volume (cc)	CI	Other Radiation Treatment
1	1st	Progression	C1-2	24	3	29.27	17.13	1.61	Once, after index instrumentation
	1st		C5-6	24	3	29.63	17.79	1.68	
	2nd	Residual	C6	20	2	26.32	12.35	1.28	
2	1st	Residual	C2	25	5	31.25	30.83	1.48	NA
	2nd	Progression	C1-3	27	3	33.75	60.80	1.10	
3	1st	Primary Control	C2	24	3	30	13.76	1.29	Twice, after index instrumentation/adjuvant SRS
4	1st	Progression	T12	21	3	25.82	6.20	1.63	Once, prior to index instrumentation
	1st		L1	21	3	26.25	26.20	1.73	NA
	1st		L2	21	3	26.04	23.50	1.52	
	2nd	Progression	L3	24	3	32	8.46	1.27	
5	1st	Residual	T11	22	1	28.2	100.67	1.23	NA
	2nd	Progression	L1	20	1	25	42.69	1.22	
	2nd		L4	20	1	25	46.14	1.16	
6	1st	Boost	T4	16	1	20.51	5.11	1.54	Once, before index instrumentation
	2nd	Residual	T4	20	2	23.80	12.56	1.28	
7	1st	Residual	T5	27	3	36.48	33.48	1.48	Once, before index instrumentation
	2nd	Residual	T5	35	5	43.75	74.08	1.12	
8	1st	Progression	L3-4	20	2	25.64	0.85	2.04	Twice, after first and second instrumentation
	2nd	Progression	C1-2	24	3	30	0.77	1.59	
9	1st	Progression	T6	27	4	35.06	21.04	1.35	IMRT, after 3^rd^ CK
	2nd	Progression	T4-6	18	1	24	34.08	1.35	
	3rd	Progression	T3	18	1	23.68	6.28	1.26	
10	1st	Residual	T8	27	3	38.57	30.75	1.86	Once, after second instrumentation
11	1st	Progression	T4	27	3	34.61	35.77	1.20	Twice, after index instrumentation as adjuvant and after 1^st^ CK
	2nd	Progression	T9-11	24	3	30	247	1.29	

Reason for SRS at the index treatment was for residual tumor in 4/11 (36%) patients and for tumor progression in 5/11 (45%) patients. An additional one (9%) patient underwent SRS initially for primary control, and one (9%) for boost treatment. Second SRS was indicated for residual tumor and progression in 3/9 (33%) and 6/9 (67%) patients, respectively. Among those requiring repeat SRS treatment, 1/8 (12.5%) underwent SRS at a distinctly different spinal segment. There were 10/11 (91%) and 7/9 (78%) patients with symptoms at first and second SRS, and 8/11 (73%) and 2/7 (29%) with subsequent improvement. Two patients were without follow-up after their second SRS treatment.

A total of 16 decompressive procedures were performed. An unstable spine was identified in 6/11 (55%) at initial presentation, and all repeat surgeries (6/6) were for decompression. Time to second surgery was median 14.5 (9-34) months. A mean 5.4 (2-10) and 3.75 (2-5) levels were treated on first and second surgery, respectively. Among the four patients who had a repeat procedure involving instrumentation, one took place at a distant spinal segment. Symptomatic improvement was experienced by 9/11 (82%) and 1/4 (25%) patients following their initial and second instrumented surgeries, respectively. Among index procedures, 5/11 (45%) patients underwent a corpectomy and 4/11 (36%) took place at a junctional level. Allograft-alone was utilized in 6/11 (54%) index procedures, as well as in 1/4 (25%) and 1/1 of second and third procedures, respectively. At the time of discharge, 14/16 (87.5%) dispositions were to home.

## Discussion

Current improvements in oncologic care will yield prolonged survival that demands recurrent considerations for instrumented stabilization and risks of revision [10]. Here we characterized the heterogeneous and complex clinical histories of patients with extended follow-up and determine that pseudarthrosis remains a prominent risk, as 27% were with radiographic evidence of lucency. However, a complete survey of their clinical courses suggests that patients with spinal metastasis tolerate aggressive surgical management with perioperative SRS, and may present with progression and urgent decompression needs that outpace competing hardware risks.

Variations in surgical approaches, radiographic follow-up and timing of adjunct radiation treatment make comparisons difficult between published studies, but our pseudarthrosis rate is consistent with the reported 0-43%. On the lower range, Harel et al. identified no instrumentation failure (0/8) with a more conservative median 14.75 Gy single fraction treatment at median 15.9 months, while Amankulor et al. reported a 2.8% (9/318) symptomatic hardware failure rate at a median 19 months, all with compression fractures perhaps related to long and/or posterior-only stabilization [11, 12]. Aside from our broader criteria, a higher pseudarthrosis rate in our series may be attributed to longer follow-up and inclusion of patients’ multiple courses of radiation, thereby capturing the lifelong pseudarthrosis rate.

Higher instrumentation failure rates were reported in earlier XRT studies where perhaps dosing regimens had not yet been optimized [8, 11]. Emery et al. noted that patients with pseudarthrosis were all with total XRT > 40 Gy, but the statistical significance of this threshold was eliminated once converted to BED [8]. The more contemporary option for SRS with higher prescription doses is largely preferable to XRT to achieve local control [1, 7, 11]. This could preserve bony integrity and preclude implant externalization, as was necessary in one of our patients and which our BED analysis supports, perhaps obviating stress-imposing extension of constructs as in one of our cases [11].

Most importantly, our review further qualifies the role of perioperative SRS despite hardware risks [13, 14]. We see that radiographic pseudarthrosis is more prominent than symptomatic failure. Time to pseudarthrosis occurred at 36, 54, and 117 months, which was longer than the median time to retreatment. The benefits to quality of life are evident in the rate of symptomatic improvements and the rate at which they discharge to home. In the same vein, Laufer et al. reported an optimistic functional status following repeat posterolateral decompression (without radiation), with 65% still ambulatory at the time of last follow-up and with unchanged rates of estimated survival [6].

Our results and prior work will help counsel patients with recurring pathology and spinal surgeries. Local recurrence is estimated to affect a quarter of patients [6, 14, 15]. Likewise, re-operation is anticipated for 25% of spinal metastasis patients. Although pseudarthrosis currently does not appear to fully correlate with symptomatic failure, there is the future risk that with prolonged survival, patients will increasingly experience pain associated with instrumentation and perioperative SRS. Post-operative radiation is known to have detrimental effects on fusion, particularly in the early post-operative period [16, 17].­ If patients are anticipated to experience multiple procedures, such as those with favorable pathologies, perhaps more frequent spinal imaging should be obtained in select populations to provide neoadjuvant SRS.

## Conclusions

Here we provide a complete longitudinal assessment of patients with instrumented metastatic spinal disease who required re-operation and re-irradiation, not captured in prior works. We suspect symptomatic pseudarthrosis rate is an overestimate of the problem given the number of patients lost to follow-up due to survival. We anticipate with the improvement of individualized therapy and increased availability for SRS treatment, future studies will offer greater follow-up and sample size for statistical assessment. While we reaffirm a genuine risk for pseudarthrosis following perioperative SRS, the associated symptoms are minimal and should not deter aggressive radiation and surgical management if with good prognosis.
